# Negotiating privacy and responsibility in digital public health: a qualitative study of the social and ethical implications of peer-to-peer health data sharing

**DOI:** 10.3389/fdgth.2026.1748259

**Published:** 2026-03-25

**Authors:** Jens Lindberg, Anna Sofia Lundgren, Malin Eriksson, Helena Lindgren

**Affiliations:** 1 Department of Social Work, Umeå University, Umeå, Sweden; 2 Department of Culture and Media Studies, Umeå University, Umeå, Sweden; 3 Centre for Demographic and Ageing Research (CEDAR), Umeå University, Umeå, Sweden; 4 School of Social Science, Södertörn University, Huddinge, Sweden; 5 Department of Computing Science, Umeå University, Umeå, Sweden

**Keywords:** datafication, digital health, ethics - care ethics, health data, health information, peer-to-peer sharing, public health, social media

## Abstract

**Introduction:**

Peer-to-peer sharing of personal health data on social media is increasingly used as a strategy to support public health goals. Such sharing is often assumed to motivate individuals to adopt or maintain healthy behaviors. However, the social and ethical implications of sharing-based interventions remain insufficiently examined. This paper offers an empirical and theoretical contribution by foregrounding the socio-technical contexts of sharing and analyzing how sharing-based interventions may drive social change. Building on these insights, it also outlines ethical considerations for researchers and stakeholders.

**Method:**

We conducted 22 semi-structured interviews with participants in a regional public health intervention in Sweden. Interviews focused on participants' experiences of receiving personal health data and their reflections on sharing such data on social media. Analysis was guided by reflexive thematic analysis and informed by theoretical perspectives on the socio-technical embeddedness of health data and sharing practices.

**Results:**

Participants understood health data as both personal and communal. Although many expressed discomfort with disclosing sensitive health information online, the peer-to-peer sharing model fostered a perceived moral obligation to share data for collective benefit.

**Discussion:**

The tension between personal boundaries and perceived communal obligations raises important ethical concerns, particularly when individuals feel pressured to share data they would prefer to keep private. Our findings underscore the need for ethical frameworks that address social pressures, consent, and the emotional dimensions of data sharing. To support sustainable and ethical public health practices, further qualitative research is essential—particularly to understand how individuals navigate obligations and risks in technology-mediated care, and how these dynamics shape values such as autonomy, well-being, and collective responsibility.

## 1 Introduction

The sharing of personal health data by laypeople on social media is increasingly being framed as an opportunity for public health (1, 2). Peer-to-peer interactions about health and lifestyle on platforms like Facebook, Instagram, and other social media systems are believed to inspire individuals to adopt or maintain healthy behaviors (3). As people spend increasing amounts of time on social media (4), and given the perceived potential of these platforms, peer-to-peer sharing interventions are now being promoted and utilized to achieve public health goals (5, 6).

Despite the growing interest in people's sharing of health data, limited efforts have been made to explore the topic (7). Existing research has almost exclusively focused on consolidating people's sharing practices, with empirical studies primarily conducted from cognitive, behavioral, or technological perspectives (6). According to Lupton (8), scholarly discussions about data and data sharing are often individualized, and “discussions of how people collect and make sense of their own data can be reduced to models of cognition or behavioural psychology.”

Health-related data sharing on social media is part of broader processes of *datafication*, where bodies and practices are increasingly being transformed into data flows that can be collected, quantified and used for various purposes (9). It generally takes two forms: narrative disclosures of personal experiences relating to health, illness, or treatment, and the sharing of clinical data such as medical test results. Although framed as public health promotion, such sharing takes place on commercial social media platforms and is sustained by networks of laypeople. Nevertheless, the social and contextual conditions underpinning this sharing remain largely unexplored, and its social and ethical implications warrant further investigation (5, 6, 10). This need is particularly pressing given that the social conditions fostered by sharing-based interventions—interventions based on the practice of sharing health-data in digital communities—may influence both individual and public health and well-being (11). To date, however, ethical discussions have largely remained within biomedical and technological frameworks (12), often shaped by the aspirations and assumptions of public stakeholders (13). Although efforts have been made to align social media-based interventions with various ethical principles (14, 15), such initiatives have often overlooked perspectives that recognize the active role these interventions can play in driving structural social change. As digitally mediated spaces for healthcare, such as apps and social media, become integral to everyday care, scholars have highlighted how social relations are being reconfigured, leading to shifts in established patterns of individuals behavior and emotional engagement (16). When health data are shared and consumed on social media, they not only influence how people approach health and lifestyle; they also help reshape shared notions of essential public services and individual responsibilities within society, particularly in relation to health and healthcare (17). From this perspective, it is becoming increasingly clear that evolving relationships and responsibilities emerging from sharing-based interventions warrant further scholarly exploration, and that they should be approached through an ethical perspective.

In this paper, we focus on social and ethical implications of public health interventions that involve, or have the potential to involve, peer-to-peer sharing of personal health information. We draw on interviews with participants in a regional public health intervention in Sweden who had recently received their personal health data as part of the program. Although the intervention did not explicitly promote peer-to-peer sharing, it was undergoing a process of digitization. We explore how participants understood their data and how they reflected on the idea of sharing it on social media. The complexity of researching digital health interventions warrants collaborations across disciplines (18). Combining insights from digital health, cultural studies, public health, and health informatics, this paper aims to make two key contributions. First, given the normative stance found in much previous research (7, 11), this paper provides a theoretical and empirical shift by focusing on the socio-technical contexts of sharing and, ultimately, the role of sharing-based interventions in driving social change. We believe this perspective offers fresh insights into the social and ethical implications of sharing-based interventions (19). Second, the paper provides reflections on ethics for researchers and stakeholders to take into account. Interventions involving peer-to-peer sharing have often been portrayed in an unequivocally positive light. Still, there is no consensus on whether or how such sharing promotes or jeopardizes health and well-being (6, 20). Moreover, digital health technologies have unintended, unforeseen, and sometimes negative consequences for individuals' health, welfare organizations, and society at large (21, 22). Consistent with prior scholarly work on ethics (23, 24), we argue that while sharing-based interventions may appear promising, their appealing potential for public health should not replace careful ethical scrutiny.

In the following, we will provide empirical and theoretical backgrounds necessary to understand and approach sharing-based public health interventions from a social and ethical perspective. In the results section, we will present two shared yet competing perspectives on data and data sharing as expressed by the study participants. Building on these findings, we will discuss the role of sharing-based interventions in driving social change. Finally, we will offer notes on ethics that we believe are important to address.

## 2 Empirical and theoretical backgrounds

As peer-to-peer sharing emerges as a strategy in public health work, sharing-based interventions occupy the intersection of at least three areas: ethics in public health, ethics in digital health, and ethics in peer-to-peer sharing of health data.

### 2.1 Ethics in public health

Public health is broadly defined as preventing disease, prolonging life, and promoting health through collective societal action (25). Unlike biomedicine's individual focus, it targets populations, requiring ethical approaches that account for how personal behaviors influence communal well-being (26, 27). Because public health interventions often shape individual choices for societal benefit, they raise enduring tensions between autonomy and collective welfare (28).

Ethical reasoning in this field is commonly organized into practice-based and theory-based approaches. Practice-based frameworks, often rooted in bioethics, prioritize actionable guidance; for example, Upshur's (29) principles of harm, least restrictive means, reciprocity, and transparency. Theory-based perspectives draw on broader moral traditions—utilitarian, Kantian, and communitarian—but have been critiqued for conceptual ambiguity. To address this, Petrini and Gainotti (30) propose personalism, emphasizing respect for persons alongside autonomy, confidentiality, equity, and fair distribution of resources.

Despite their differences, these models converge on core values such as autonomy, freedom, well-being, and the public interest, highlighting that ethical public health practice must be context-sensitive and protective of personal integrity. Yet these frameworks remain limited in fully resolving the inherent tension between individual rights and population-level goals.

### 2.2 Ethics in digital health

Digital technologies now permeate healthcare, supporting documentation and communication, delivering interventions, and self-monitoring through apps, platforms, and social media. Ethical debates in digital health often draw on biomedical and principalist frameworks (31), though many values emerge through technological and legal discourse. Regulation increasingly aims to ensure equitable access and compliance with privacy and safety standards (32), while legal protections for health data reflect ethical concerns such as privacy, justice, and trust (33). For digital tools aimed to change behavior, additional principles, including transparency and user control, help safeguard against coercion or manipulation (34).

Ethical considerations, however, diverge depending on a technology's purpose and context. Tools embedded within healthcare systems operate under public mandates, whereas those integrated into commercial social media platforms operate outside them and are driven by data-monetization business models. Platforms such as Facebook and Instagram profit from health-related data through targeted advertising, creating a hybrid environment in which commercial infrastructures are used for public health purposes. This raises important questions about whether commercial actors should be held to ethical expectations comparable to those in healthcare. Recent guidelines therefore emphasize as user agency, privacy, data governance, and transparency (35, 36), reflecting recognition of the ethical complexities at the intersection of public and commercial interests. These complexities underscore the need for further research into how public health actors encourage individuals to share their health data within such environments.

### 2.3 Ethics in peer-to-peer sharing of health data

Ethical discussions about sharing health data on social media have largely centred on privacy and personal integrity. Although no comprehensive frameworks exist for ethics specific to peer-to-peer sharing (15), research shows that people are generally willing to disclose health information to secondary or tertiary parties as long as anonymity is protected (37). Yet concerns about exposure, privacy loss, and negative judgment persist, even as users describe benefits such as empathy, motivation, and enhanced self-worth (38–40). As a result, sharing health information online can feel both reassuring and unsettling (41).

Despite viewing health data as highly personal, many users share it routinely and voluntarily, often with minimal protective strategies (42, 43). This paradox reflects a broader pattern in which disclosures are motivated by perceived personal or collective benefit, while they also occur unintentionally through ordinary platform use (7).

These findings illustrate that sharing practices are shaped by socio-technical conditions in which social norms and platform design influence what, how, and why people disclose (8). Ethical perspectives should therefore treat health data sharing not as isolated, autonomous decisions but as situated, negotiated practices embedded in specific contexts (38). This underscores the need for ethical frameworks that account for the socio-technical dynamics structuring participation in social-media-based health interventions.

### 2.4 Theoretical framework

To address social and ethical implications of peer-to-peer health data sharing interventions, we draw on socio-technical perspectives from critical health and media studies, highlighting how technologies are inseparable from the social practices, norms, values, and power relations that shape their use. We conceptualize health data and its sharing as temporary materializations of individual and communal identities, shaped by social norms and technological affordances—the capabilities for action that emerge from the relationship between users and technology (8). In digital contexts, identities blur boundaries between the social and the technological (44), with data sharing acting as a form of double-embodiment where individuals extend themselves into digital spaces, while technologies become embedded in their lived experiences (45).

Lupton's (8) notion of “lively data” not only emphasizes the constant generation and recombination of digital data through people's online interactions, but its “implications for human life opportunities and livelihoods”, hence capturing the deep entwinement of actors and contexts in making sense of data. Through repeated sharing on social media, personal health data becomes active in shaping notions of public goods, social commons, and responsibilities (46). Thus, data sharing is not merely a response to perceived needs but a socio-technical practice that reconfigures values, identities, and institutions.

Technological affordances, as described by Norman (47), refer to the possibilities for action that arise in the interaction between users and technology. These affordances offer cues about intended use, and within social media interventions, they intersect with users' motivations and intentions to shape behavior (48, 49). Such interventions often aim to align individuals with normative health values, potentially sidelining alternative perspectives. As Shaw and Donia (13) argue, digital health technologies are designed to promote specific normative goals.

From this standpoint, we advance the core hypothesis that the socio-technical interplay of peer-to-peer sharing, platform infrastructures, and user practices transforms prevailing understandings of healthcare roles and responsibilities. Attending to these dynamics is crucial for developing ethical frameworks attuned to the realities of digital public health interventions (31, 50).

## 3 Materials and methods

The following sections outline the selection of study participants and address research ethics. We then describe the methods used to collect the research material and the approaches applied in its analysis.

### 3.1 Selection

This study draws on 22 semi-structured interviews with individuals who recently participated in the Västerbotten Intervention Program (VIP), a long-standing public health initiative in northern Sweden. The VIP aims to monitor personal health and encourage lifestyle changes through regular health examinations offered at ages 40, 50, and 60. Participants undergo assessments of physical activity, diet, tobacco and alcohol use, BMI, waist circumference, blood pressure, blood glucose, cholesterol, and self-reported health. Results are then communicated via a star-shaped visualization (51) and discussed in a “health dialogue” with a nurse to support behavioral change. The visualization that had previously existed on paper was to be digitized, creating interest in knowing how people felt about sharing their health data within online communities (52).

The study sample included 13 women and nine men, aged approximately 40, 50, or 60, living in both urban and rural areas. Participants were selected using a theoretical sampling approach (53) to ensure diversity in age, gender, living conditions, and medical history and capture diverse and potentially significant characteristics and experiences. Based on those premises, participants were also selected for the study regardless of their previous experiences of gathering and sharing personal information on social media. All participants used social media, primarily Facebook and Instagram, with messaging apps like WhatsApp and Messenger used in more intimate social circles. None reported using disease-specific online platforms.

To recruit participants, we obtained a contact list from the VIP registry. Recruitment was conducted independently of healthcare staff and involved four phases of outreach. Selected individuals received an information letter by post, followed by a phone call from the first author to explain the study and arrange interviews. Of the 30 contacted, 22 agreed to participate, introducing an element of self-selection. This empirical foundation enabled the exploration of participants' experiences and perceptions of health data and its potential sharing on social media, contributing to broader discussions on socio-technical dimensions and ethics in sharing-based public health interventions.

Given the sensitive nature of health and social media use, ethical considerations were paramount. The study adhered to guidelines from the Swedish Research Council (54, 55), ensuring confidentiality and minimizing identification risks. Therefore, no names or detailed personal information are disclosed. The study was approved by the Swedish Ethical Review Authority (Dnr: 2019-02924; 2020-02985).

### 3.2 Research materials

The research was conducted between 2020 and 2021. Initially, all interviews were planned to be in person, but due to COVID-19 restrictions, most were conducted remotely via Zoom. The first author carried out around two-thirds of the interviews, with the remainder conducted by the second author. The first five interviews were held face-to-face before restrictions were imposed. During Zoom sessions, participants could choose to use their cameras, though only audio recordings were made.

The interview format was adapted in line with pandemic-related adjustments to qualitative methods (56), prioritizing brevity and specificity over the depth typical of in-person interviews (57). In-person interviews lasted up to two hours, while remote sessions via Zoom averaged just over one hour, with the longest at one hour and forty minutes. No follow-up interviews were conducted. Recruitment, interviewing, and preliminary analysis continued until authors one and two agreed that sufficient data had been gathered (58). All interviews were transcribed verbatim, with written consent for in-person sessions and recorded verbal consent for Zoom interviews.

Interviews were structured around five central themes: everyday use of social media platforms; attitudes towards sharing and accessing health-related information; the influence of social media on health management; definitions and communication of personal health data; and privacy concerns. Participants also reflected on their experiences with the VIP, including the health examination and follow-up dialogue with a nurse.

The interview guide was developed through five interrelated steps (59). First, the suitability of semi-structured interviews was assessed. Second, existing methodological and thematic knowledge informed the guide's development. Third, authors one and two drafted a preliminary guide. Fourth, authors three and four validated the guide, which was then tested during initial interviews. Finally, the guide was refined iteratively, incorporating emerging themes for subsequent interviews.

Although later interviews followed similar lines of questioning, the depth and direction of responses varied depending on participants' trajectories. This variation is a recognized strength of semi-structured interviews, allowing both convergence and divergence in responses (60). Such diversity enhanced the study's scope and analytical relevance (58). For instance, participants' recent engagement with the VIP helped them recall similar experiences while elaborating on different aspects of health data and sharing practices. The semi-structured format also enabled participants to explore topics beyond the predefined themes, contributing to richer discussions (61). This flexible approach supported the study's aim to understand nuanced perceptions of health data and its sharing within socio-technical contexts. No follow-up interviews were conducted. As an exploratory qualitative study, we prioritized depth over longitudinal breadth, and the single-interview format provided sufficiently rich accounts within the project's scope. Given the potential sensitivity of discussing personal health data and sharing practices, re-contacting participants risked causing discomfort. We therefore chose not to pursue additional interviews.

The COVID-19 pandemic introduced limitations to the interview process, particularly in remote formats. Zoom interviews were generally shorter and sometimes hindered deeper engagement with sensitive or abstract topics (56, 57). To mitigate this, we clarified complex questions and encouraged inclusive dialogue. Despite these constraints, participants shared rich insights into their health and social media use, discussing both specific medical data (e.g., blood pressure, cholesterol) and broader lifestyle factors (e.g., diet, exercise). They also reflected on abstract themes such as their relationship to health data and attitudes toward sharing. Perceptions varied: what one participant considered sensitive, others did not. However, most agreed that while health data is personal, the affordances of social media and perceived benefits of sharing made disclosure seem reasonable. This tension between privacy and perceived utility underscores the complexity of health data sharing in digital environments.

### 3.3 Analytical approach

The research team brings together perspectives from Social Work, Computing Science, and Ethnology. These backgrounds shape how we understand digital health practices; Social Work foregrounds care and vulnerability, Computing Science highlights data and technological infrastructures, and the ethnological training of the first two authors brings attention to lived experience and cultural context. Our interdisciplinary backgrounds also fostered a shared focus on the socio-cultural dimensions of datafication as they emerged in people's reflections. Hence, we analyzed transcripts not only for expressed attitudes, but for how participants' narratives revealed the entanglement of technological affordances, social media norms, and public health ideologies. The analysis was guided by Braun and Clarke's (62) reflexive thematic approach and informed by socio-technical theories on the embeddedness of health data and sharing practices (8, 45). The analytical work was also shaped by three conceptual lenses—affordance, double-embodiment, and lively data—which we used not as preset coding categories but as sensitizing concepts that helped us explore participants' ways of relating to their data.

In the first step, the first and second authors read, interpreted and coded the transcribed research material, focusing specifically on instances when participants described and defined their health data or expressed feelings about their data. By looking for shared meaning-making about data and data sharing, the first and second authors separately generated codes relevant to the research question. In the second step, the first and second authors reviewed, synthesized and refined the codes by negotiating and finding a shared understanding and vocabulary for the research material. This process also served as an internal validation, which, besides critically discussing the codes, included finding mutual and productive ways to pursue the inquiry.

In the third step, we moved from codes to themes by grouping refined codes into broader patterns of meaning. Through iterative discussion, the first and second authors identified two contrasting clusters regarding ownership of personal health data—one emphasizing personal control and ownership, the other viewing health data as a communal resource—which subsequently formed the basis for two overarching and partly opposing themes. These themes were then refined and named in line with the paper's focus on personal data ownership (see Table 1). The analysis was an iterative process, where authors one and two moved between the empirical material and conceptual ideas to generate meaningful analytical concepts (63). A key part of this process involved collaboratively reviewing codes and themes, critically reflecting on assumptions and potential biases (64). In the fourth step, the two overarching themes were empirically described and illustrated, showing how participants' understandings of health data and sharing were both contrasting and interconnected. Presenting the themes in this way helped to capture the complexity of participants' perspectives and the socio-technical dynamics underpinning their engagement with health data. Here, the three analytical concepts supported interpretation, enabling us to articulate how participants' sense-making was shaped by the perceived functions of the data (affordances), their embodied relations to data (double-embodiment), and the ways their data lived socially beyond the self (lively data).

**Table 1:** Coding scheme.

Quotes	Codes	Overall themes
“A weird sense of pride that finally (…) having a waist size that's ‘approved’”	Health data as a personal characteristic	Health data as personal property
“I'd stopped smoking and the results were good, so I was happy”	“Good” and “bad” data	
“I had too much blood fat (…) and that's not good”		
“I think [health data] is more of a personal thing”	Sharing as self-exposing	
“I don't have to brag about it. [That includes] my [medical] results”	Sharing as boasting	
“So you motivate each other that way”	Sharing health data can help motivation	Health data as communal property
“I would share [health data] if it became a motivation for others”	Helping others	
“It is mine [health data]. It might seem a little selfish”	Not sharing is selfish	

To deepen the empirical analysis and more explicitly situate the findings within ongoing ethical debates, the fifth step involved theorizing how participants' understandings of data and sharing were shaped by broader social and ideological contexts in health and public health. This analytical stage entailed comparing participants' accounts with existing scholarship on public health ethics, digital health, and practices of social media sharing. By engaging these bodies of literature, we explored points of alignment between participants' perspectives and established conceptual debates, thereby illuminating how the growing use of sharing-based interventions may contribute to shifting collective understandings of individual responsibilities in public health. In a final step of the inquiry, we outlined ethical considerations and research agendas we believe are essential for responsibly integrating peer-to-peer sharing into future public health initiatives.

## 4 Results

Two dominant understandings of health data and its sharing emerged from the analysis of the interviews. First, participants viewed medical results as personal attributes, with reluctance to share data depending on whether they perceived it as “good” or “bad”. Second, data was also seen as communal property, where sharing was considered helpful to others pursuing healthier lifestyles. These contrasting yet interconnected views reflect how notions of ownership and responsibility are shaped by both personal values and socio-technical contexts.

### 4.1 Health data as a personal property

When discussing health-related data, participants tended to classify their results as either “good” or “bad”. This valuation was strongly shaped by biologized and medicalized understandings of health and lifestyle. Many described the VIP as a medical “check-up” that positioned the body as something that could only be accurately assessed by clinical expertise: “It's like a check-up for blood values… like having your car inspected” (Participant 2). Participants also described their satisfaction with their health primarily in relation to these medical results rather than their own subjective feelings of well-being:

Well… I was satisfied when I left [the medical examination]. I'd stopped smoking and the results were good, so I was happy (Participant 2).

Following a similar logic, results that did not meet the medical standards defined in the VIP were conceived as bad and described to affect wellbeing:

I guess it's good to have [the medical examination] but you *do* find out about results that you'll have to work on [laughing] (…). Then maybe you don't feel at your best [laughing]! (Participant 11).

At times, participants questioned the medical basis for defining “good” and “bad” data. Nonetheless, medicalized perceptions appeared deeply internalized, making medical results a foundation for evaluating not only health and health data but also themselves:

I was just on the edge of what was “ok” (…) It was a… weird mix of feelings. In part, when considering the internalized shame that I've lived with, to have a body that has “volume” at the middle, but also a weird sense of pride that finally, at the age of forty, having a waist size that's “approved” (Participant 19).

This translation of health data into personal traits contributed to participants' reluctance to share health data on social media. Consistent with previous empirical findings, most participants did not want to share “poor” results. Many also expressed concerns about appearing self-promoting, meaning that even sharing “good” results was seen as potentially boastful:

That's a bit boastful I think. (…) I'm not a boastful person—“action speaks louder than words”. I can show people what I do but I don't have to brag about it. [That includes] my [medical] results (Participant 10).

Overall, participants seemed reluctant to portray themselves negatively for fear of provoking adverse responses. They anticipated judgement, especially for “poor” results, and expected harsh or moralizing comments. In such cases, the personal nature of data became the decisive factor motivating their reluctance to share:

Nope! (…) I think [personal health data] is more of a personal thing. (…) Well, people [on social media] would probably say “Have more exercise and less candy” and that kind of thing [laughing] (Participant 9).

Reluctance to share thus stemmed both from concerns about negative reactions and from the sense that health data were tightly linked to the individual. “Bad” data risked reflecting a flawed lifestyle, while “good” data risked reflecting an undesirable personality (boastful, prideful). This framing reinforced the idea that health data function as a form of personal property closely tied to identity.

There were, however, competing and contrasting interpretations. Within the context of social-media-based interventions, some participants also described health data as a form of communal property: something shared, compared, and made meaningful collectively. This understanding of personal information as socially embedded motivated participants to reinterpret risks and occasionally downplay concerns, making sharing seem like a contribution to a collective practice rather than solely an exposure of the self.

### 4.2 Health data as a communal property

While medicalized and individualized perceptions were clearly dominant, participants' understandings of health data were also shaped by what they believed their data could *do*. Despite concerns about risks, many expressed a willingness to share personal health information if it could support others in their health journeys. Some were already doing so; for example, one participant regularly posted health-related updates to “try to motivate” friends and family (Participant 13). Others, though not currently sharing such information, said they could imagine doing so for similar reasons, viewing their health data as potentially beneficial to others:

Maybe like: “I've done my share now. This is what I've accomplished today”. To [post the implicit question]: “How about you? Have you done your exercise?” [laughing]. So you motivate each other that way (Participant 5).

Participants noted that excellent medical results could inspire others to set health goals, but also felt that “bad” results might be even more valuable because they were more relatable and supportive. They were generally open to sharing such data, within contexts where recipients faced similar circumstances or where mutual encouragement could develop. This reflects a nuanced view in which both positive and negative results are seen as meaningful contributions to collective health efforts:

I wouldn't talk about bad medical results publicly, but if it was a *group*, it would be more like a… well, I would think that it'd help a lot, because you've got the same [problems] and can support each other (Participant 4).

While biologized, medicalized, and individualized understandings of health data were prevalent, participants also expressed views shaped by collective ideals. Individually, “good” health data was often defined through established medical standards, with participants evaluating their results based on proximity to normative health benchmarks. Yet in social media contexts, these definitions shifted, with participants reinterpreting their data through its potential value to others. Even “bad” results were sometimes considered useful if they fostered encouragement or solidarity. This reframing positioned personal health data not solely as private property but as a communal resource with social utility.

Such perspectives revealed an ongoing negotiation between individual ownership and communal responsibility. Participants often shifted between these positions, at times describing their reluctance to share as “selfish”, even while insisting that their data were inherently personal: “It feels like it's my business [laughing]. It is *mine.* It might seem a little selfish, but…” (Participant 2). This tension illustrates how peer-to-peer sharing interventions can prompt individuals to reconsider the boundary between private and public, and how personal health data may be reimagined as part of a collective effort in public health.

Overall, the perceived value of sharing health data on social media evoked a sense of moral duty among participants. Many believed that making their personal health information public could meaningfully support others, by raising awareness, fostering understanding, or contributing to broader health efforts. This belief prompted some to reframe their data as having communal value rather than being solely personal. Yet this moral impulse was consistently tempered by concerns about privacy and risk. Despite acknowledging the potential societal benefits of sharing, most participants felt uneasy about disclosing their data, citing fears of misuse, loss of control, and unwanted exposure. The result is a central ethical tension: a pull toward altruistic sharing motivated by perceived responsibility, countered by a pull toward self-protection rooted in personal vulnerability. While collective benefit could inspire willingness to share, it did not override the deeply personal risk assessments each participant had to navigate.

## 5 Discussion

The analysis revealed two dominant, interrelated understandings of health data and its sharing. First, participants viewed their medical results as *personal property*—private and closely tied to individual identity. These results were often judged as “good” or “bad”, and reluctance to share them publicly on social media was linked to how closely they aligned with normative health standards. Second, health data was also seen as *communal property*, with the potential to support others in their health journeys. In this view, even “poor” results were considered “good data” if they could motivate or help others, aligning with broader public health goals.

Together, these perspectives reflect shared yet competing understandings of health data: private and sensitive, yet also socially valuable. Despite concerns, participants were generally more inclined to share their data than to withhold it. The context of public health interventions fostered a sense of collective responsibility, drawing participants into social media spaces where technological affordances shaped their perceptions of data and obligation.

### 5.1 Performing altruism and good citizenship

The tendency to view private, even unfavorable, health information as communal property raises ethical questions about how interventions normalize social media sharing in public health. As in other areas of life, digital engagement is increasingly framed as essential for managing personal and collective wellbeing (65). Individuals are expected to be digitally included and active. In line with this, advocates of sharing-based interventions argue that sharing health data online reflects altruism and care for others (66, 67). These trends warrant critical reflection on the ethical implications of digital participation in public health.

Rose (68) argues that since the 20th century, Western politics have increasingly governed populations in the name of health and well-being. Through “ethnopolitics”—the regulation of life and its ideal forms—individuals internalize societal norms, constraining personal freedoms and creating perceived obligations. Thus, peer-to-peer sharing interventions, while seemingly medical or epidemiological, are also deeply ideological and moral in nature, shaping how health is understood and enacted. Felt (69) highlights how socio-technical imaginaries in digital health blur boundaries between patient and citizen roles, forming a hybrid “digitally informed citizen-patient” with a moral duty to engage with health data [see also (70)]. Fotopoulou (46) consequently argues that sharing health data online has become a ritual of moral citizenship, where self-disclosure is framed not as vanity but as altruism. She further suggests that such sharing fosters a sense of productivity, contributing to public good.

In our study, participants often described feelings of productivity linked to acquiring and sharing health data to help others. These sentiments reflected shared understandings of public responsibility akin to Felt's notion of citizenship, shifting perceptions of health data from private to communal; the act of disclosing medical results, whether “good” or “bad”, was reframed as a contribution to public health. This illustrates how digital health practices are not only technical or medical but also deeply ideological, embedding moral expectations into everyday behaviors and reshaping notions of responsibility and care.

### 5.2 Public health by public exposure

Ajana (71) argues that as personal health data becomes increasingly collected and shared, it is transformed into normative facts that generate value for health actors by reinforcing ideals about health and lifestyle. In sharing-based interventions, communal data supports public health agendas by promoting these norms. Crucially, such influence depends on individuals engaging with their data collectively, sharing personal health information on social media to represent and reinforce normative standards. While many participants in our study supported sharing both “good” and “bad” data to help others, this often came with feelings of overexposure and risk. Their willingness to promote sharing despite personal hesitation suggests that such interventions cultivate new social responsibilities—namely, exposing personal data for the public good.

The online collection and sharing of health data is a relatively recent phenomenon, rooted in subcultures like the “quantified self” and “self-tracking” movements of the early 2000s (72). While leveraging these practices for public health is innovative, the adoption of social media infrastructures by public actors marks a significant shift. It extends public health efforts into vast, unbounded digital spaces, using individuals' bodies and emotions in new ways. Historically, personal health data was collected, anonymized, and used by authorities to inform public guidance. In contrast, peer-to-peer sharing exposes personal data to potentially unlimited audiences, including strangers. While this communal approach may support public health goals, it also intensifies structural challenges linked to social media and raises new ethical concerns. These developments call for careful ethical scrutiny, particularly regarding privacy, consent, and the shifting boundaries between public and private life.

### 5.3 Notes on ethics

Emerging technologies in the Western world are often portrayed as having limitless potential with minimal risk (73), a narrative that dominates digital health policy and research. Within this context, peer-to-peer sharing interventions are framed as promising innovations, with personal health data seen as an untapped resource. However, this optimistic discourse tends to obscure ethical concerns, particularly those arising from structural social change.

This paper identifies several critical tensions in sharing-based public health interventions. The first is the conflict between public health goals and individual autonomy, a tension commonly addressed within the ethics of public health. However, to fully understand this conflict, it is necessary to incorporate ethical insights from the fields of critical digital health and peer-to-peer health data sharing. By taking these perspectives seriously, we were able to identify two additional tensions. The second involves the integration of ethical principles into commercial infrastructures governed by business logics, which raises concerns about the commodification of health and the erosion of trust. The third tension arises from the mismatch between social media sharing norms and individuals' desire for privacy and control over their personal data. These insights reveal the need for ethical frameworks that reflect the complexities of digital environments and the social dynamics that shape participation in data sharing practices.

Rose's (74) “preventive paradox” highlights how population-level benefits may offer little, or even harm, to individuals. Our findings suggest that social media-based health interventions risk compromising values such as autonomy, transparency, reciprocity, privacy, and equality (15, 29, 30). While research on the downsides of sharing-based interventions is limited (10), privacy concerns are prominent, with calls for tailored privacy protections (75), and warnings that digital health may exacerbate inequalities (76). Negative perceptions of sharing can deter participation, limiting access to public health initiatives (40). Moreover, removing individuals from clinical contexts risks exposing them to misinformation (4, 77, 78), with social media users often less informed and less likely to verify health information (79).

Platforms like Facebook and Instagram host vast, unstructured health data flows shaped by algorithms prioritizing commercial interests. These biases may stigmatize groups and undermine public health goals (80). Outsourcing health promotion to such systems shifts interpretive responsibility to laypeople, who are expected to both produce and consume health content.

Our analysis suggests that sharing-based interventions can create perceived obligations to engage in public health promotion via social media. Participants felt compelled to share personal data publicly, despite discomfort. This emerging ethical concern is driven by cultural imperatives, commercial platform design, and public health strategies. Yet, it remains largely unaddressed in mainstream ethical frameworks. We argue that new approaches are needed to critically assess the implications of peer-to-peer sharing in public health.

As digital technologies become embedded in everyday life, scholars urge attention to how shared practices and ethical norms evolve (81). In digital health, peer-to-peer sharing interventions can foster solidarity, yet also provoke discomfort, risk, and exposure. The rise of AI in medicine has intensified debates around anonymized personal data, with McKay et al. (82) noting that individuals retain emotional and social ties to their health information—even when deidentified. This tension between moral duty to share and personal vulnerability underscores the need for ethical frameworks that reflect lived experience.

Emerging obligations to share, shaped by platform design and public health imperatives, must be critically examined (83). Scholars advocate a shift from abstract principles to procedural ethics better suited to digital contexts (31), warning that generic ideals may obscure systemic inequities (50, 84). Ethics, as relational (85, 86), must account for how digital sharing generates new social responsibilities. To ensure ethical and sustainable public health practices, future qualitative research is essential—particularly to explore how individuals navigate the balance between collective benefit and personal risk in technology-mediated care.

Our study explored diverse experiences of social media use and health data sharing. While the methodology generated rich conceptual insights, the qualitative design and limited number of participants present constraints for transferability. It is likely that older adults or individuals who do not use social media at all would articulate different experiences and reflections. Moreover, care relations within Nordic welfare contexts have historically been characterized by equity and mutual trust (87). If peer-to-peer sharing is to be incorporated into future public health interventions, additional empirical research is needed to understand how different populations experience, perceive, and respond to sharing personal health data online, including those embedded in other welfare contexts. Attending to these situated experiences is essential for developing inclusive, ethical, and effective public health strategies.

## 6 Conclusions

As part of broader processes of datafication, peer-to-peer interactions about health and lifestyle on platforms such as Facebook and Instagram are increasingly framed as opportunities for advancing public health (1, 2). Yet this trend demands closer scrutiny, particularly with respect to the social and ethical implications that arise when public health interventions encourage the sharing of personal health information between laypeople. This study, based on participants' reflections on peer-to-peer sharing in relation to a recent public health intervention, suggests that such interventions may create perceived obligations among laypeople to disclose personal health data on social media. While many participants were willing to share, they also expressed discomfort and concern about the risks involved. Sharing data that individuals do not genuinely wish to disclose raises ethical issues that remain understudied. Future research should explore how structural social changes influence experiences and perceptions of sharing, and how these relate to core ethical values in public health, such as autonomy, well-being, and collective interest. Public stakeholders must recognize the ethical implications of these shifts. For policymakers and managers, this means exercising caution when promoting structural sharing initiatives. For healthcare practitioners, it involves understanding individuals' preferences before recommending social media as a tool for health promotion. Crucially, future interventions must avoid exploiting perceived obligations, ensuring that individuals retain control over what health data they choose to share. Given the normative push toward integrating personal social media practices into public health, we argue for deeper ethical reflection, particularly on the shifting boundaries between private and public, and the implications for autonomy, consent, and responsibility.

## Data Availability

The datasets presented in this article are not readily available because based on the ethical principles on confidentiality provided by The Swedish Research Council (2017, 2024), unauthorized persons are not allowed to access the research material. For further questions, contact Corresponding Author. Requests to access the datasets should be directed to jens.lindberg@umu.se.
